# Apparent diffusion coefficient and intravoxel incoherent motion-derived true diffusion serve as early non-contrast longitudinal biomarkers of neoadjuvant chemotherapy response

**DOI:** 10.1038/s41598-026-51568-x

**Published:** 2026-05-21

**Authors:** M. F. A. Kamis, K. Rahmat, M. T. Ramli Hamid, F. Fadzli, F. I. Rozalli, K. W. Ng, N. A. Mohd Taib, J. H. D. Wong

**Affiliations:** 1 Present Address: Department of Biomedical Imaging, Faculty of Medicine, University Malaya Research Imaging Centre, University Malaya, Lembah Pantai, Kuala Lumpur, 50603 Malaysia; 2 Department of Radiology, Faculty of Medicine, University Teknologi MARA, Sg Buloh, Selangor Malaysia; 3 Faculty of Medicine and Health Sciences, UCSI University, Persiaran Springhill, Port Dickson, 71010 Negeri Sembilan Malaysia; 4 Department of Surgery, Faculty of Medicine, University Malaya, Lembah Pantai, Kuala Lumpur, Malaysia

**Keywords:** Diffusion-weighted imaging, Intravoxel incoherent motion, True diffusion, ADC, Breast cancer, Neoadjuvant chemotherapy, Non-contrast MRI, Tumour response, Cancer, Breast cancer, Cancer imaging

## Abstract

Locally advanced breast cancer (LABC) frequently requires neoadjuvant chemotherapy (NAC), and early, non-contrast imaging biomarkers are of increasing clinical interest for monitoring treatment response. Diffusion-weighted imaging (DWI) and intravoxel incoherent motion (IVIM) MRI provide quantitative measures of tumour microstructure without gadolinium administration. To evaluate whether the apparent diffusion coefficient (ADC) and true diffusion (Dt) derived from IVIM can serve as early non-contrast imaging biomarkers of NAC response in patients with LABC. Fourteen women with biopsy-proven LABC underwent MRI at baseline (T0), after the first NAC cycle (T1), and after the third cycle (T2). ADC was calculated from b = 0 and 1000 s/mm², while Dt, Dp, and f were obtained using a full bi-exponential IVIM model incorporating all b-values (0–1000 s/mm²). Only four patients completed all three MRI timepoints. Tumour diameter and diffusion parameters were compared across time. At baseline, ADC and Dt were significantly lower in malignant lesions compared with contralateral fibroglandular tissue (*p* < 0.01). Following NAC, mean tumour ADC increased by 27.2% at T1 and 39.5% at T2, accompanied by progressive tumour size reduction (50.9% at T2). Dt demonstrated a similar upward trend. Perfusion-related IVIM parameters (Dp and f) showed no consistent differences. ROC analysis demonstrated high discriminatory performance of ADC for differentiating invasive ductal carcinoma from normal tissue (AUC = 1.00), although this finding must be interpreted cautiously given the small sample size. ADC and Dt showed early increases following NAC that paralleled reductions in tumour size, supporting their potential as practical, contrast-free imaging biomarkers of treatment-related microstructural changes. However, the limited number of complete longitudinal datasets (*n* = 4) and the pilot nature of the study require cautious interpretation. Larger prospective studies are needed to validate these findings.

## Introduction

Locally advanced breast cancer (LABC) often requires neoadjuvant chemotherapy (NAC) to reduce tumour burden, enable surgical resection, and improve clinical outcomes^1,2^. Early assessment of treatment response is clinically important, yet conventional imaging modalities such as mammography and ultrasound have limited sensitivity for detecting microstructural changes that precede observable size reduction^3,4^. Dynamic contrast-enhanced MRI (DCE-MRI) is currently the widely used reference standard for NAC monitoring^5,6^; however, its reliance on gadolinium contrast limits its utility in patients with renal impairment, gadolinium allergy, or those requiring repeated imaging where cumulative contrast exposure is a concern^7,8^.

Diffusion-weighted imaging (DWI) offers a non-contrast alternative by providing quantitative insights into tumour cellularity through the apparent diffusion coefficient (ADC)^9^. Reductions in cellular density following effective chemotherapy typically result in increased ADC values, a phenomenon well-documented in breast cancer^10–12^. Intravoxel incoherent motion (IVIM) MRI extends conventional diffusion analysis by separating true molecular diffusion (*D*_*t*_) from perfusion-related pseudo-diffusion (*D*_*p*_) and perfusion fraction (*f*), potentially offering a more detailed characterisation of the tumour microenvironment^13,14^.

While tumour size reduction remains the conventional indicator of treatment response, it may not fully reflect early microstructural changes occurring at the cellular level. In particular, tumour shrinkage may lag behind biological response, and some lesions may demonstrate delayed size reduction despite treatment effect. Diffusion-based imaging offers the potential to detect these early changes by quantifying alterations in tumour cellularity and tissue architecture. Therefore, diffusion-derived parameters such as ADC and IVIM metrics may provide complementary information to size-based assessment, particularly in clinical scenarios where contrast-enhanced imaging is limited or contraindicated.

Despite promising early reports, the clinical value of IVIM parameters in breast cancer remains uncertain, partly due to variability in acquisition, modelling, and small sample sizes^15,16^. Furthermore, distinguishing between benign and malignant lesions and predicting pathological response using IVIM remains an active area of investigation^17,18^. Given the growing need for reliable non-contrast biomarkers, there is continued interest in evaluating the feasibility and performance of ADC and IVIM parameters during NAC^19,20^. This pilot study aims to assess longitudinal changes in ADC and IVIM-derived diffusion parameters in women with LABC undergoing NAC and to explore their potential as early imaging indicators of treatment-related microstructural response.

## Methodology

### Study design and participants

This prospective pilot study recruited fourteen women with biopsy-proven locally advanced breast cancer (LABC) scheduled to undergo neoadjuvant chemotherapy (NAC) at University Malaya Medical Centre between November 2015 and November 2017. Inclusion criteria were female patients aged ≥18 years with histologically confirmed stage IIB–IIIC disease, including multifocal or multicentric presentations. Patients with contraindications to MRI, prior chemotherapy, distant metastasis, or contralateral malignancy were excluded. The study was approved by the Medical Ethics Committee of University Malaya Medical Centre (Ethics ID: 20163 − 2221) and adhered to the Declaration of Helsinki. All participants provided written informed consent. All patients received a standard six-cycle anthracycline–taxane regimen. MRI scans were scheduled at three timepoints: baseline (T0, *n* = 14) after the first NAC cycle (T1, *n* = 14) and after the third cycle (T2, *n* = 4). Attrition at T2 was due to treatment-related toxicity or patient withdrawal, and analyses were performed using all available datasets at each timepoint.

### MRI acquisition

**Fig. 1:**
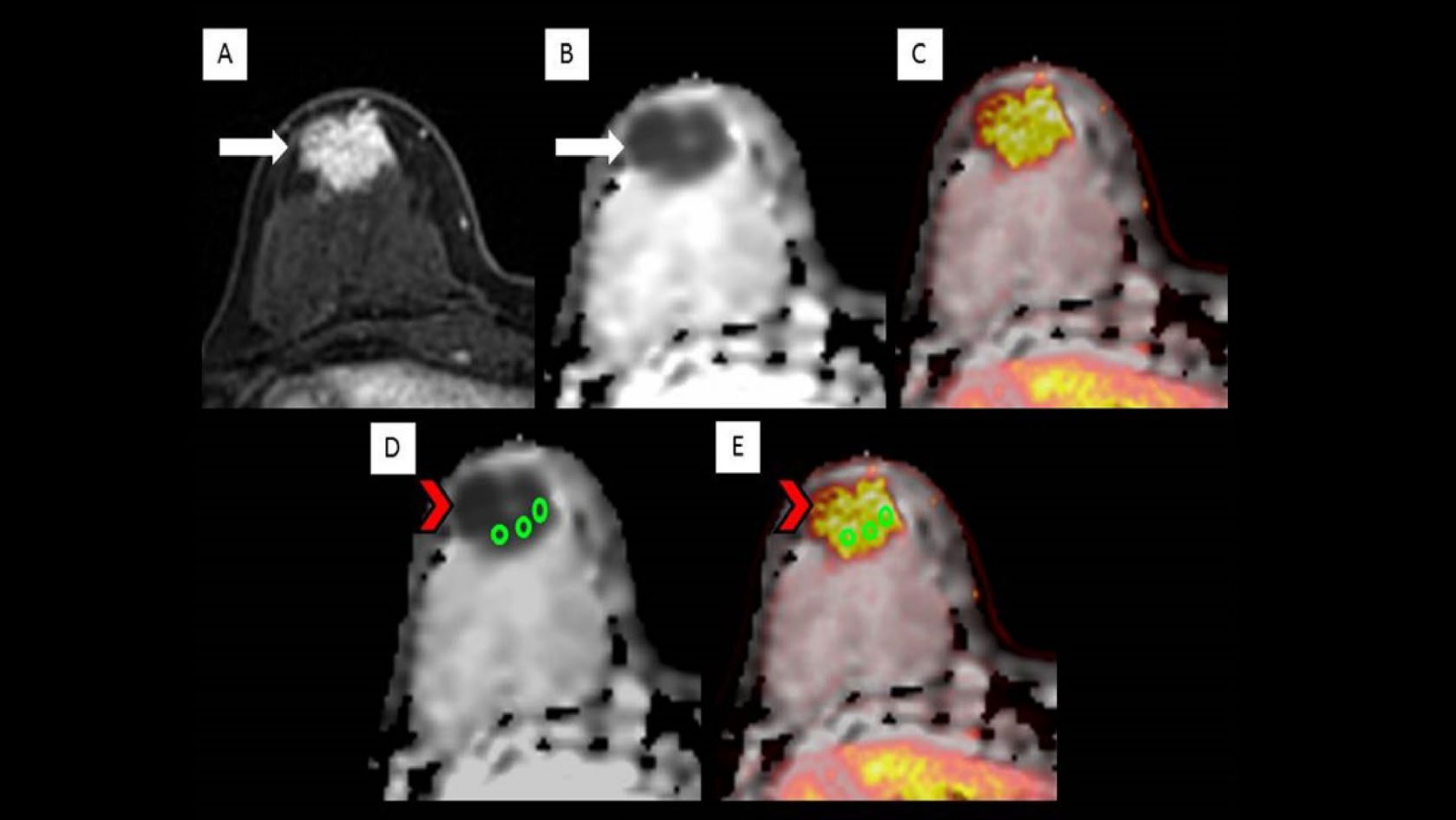
ROI placement for apparent diffusion coefficient (ADC) measurement in invasive ductal carcinoma (IDC). **(A)** Early-phase dynamic contrast-enhanced MRI (DCE-MRI) demonstrating tumour enhancement. **(B)** Corresponding ADC map showing restricted diffusion within the lesion (arrow). **(C)** Fusion of subtraction DCE-MRI and ADC map to guide ROI placement. **(D)** Single manually drawn region of interest (ROI) placed on the ADC map, avoiding necrotic or cystic components. **(E)** Fusion images confirming ROI placement within viable tumour tissue. A low-intensity region on the ADC map (arrowhead) demonstrates reduced enhancement on DCE-MRI and was excluded from analysis, consistent with necrotic change. ADC = apparent diffusion coefficient; DCE-MRI = dynamic contrast-enhanced magnetic resonance imaging.

**Fig. 2:**
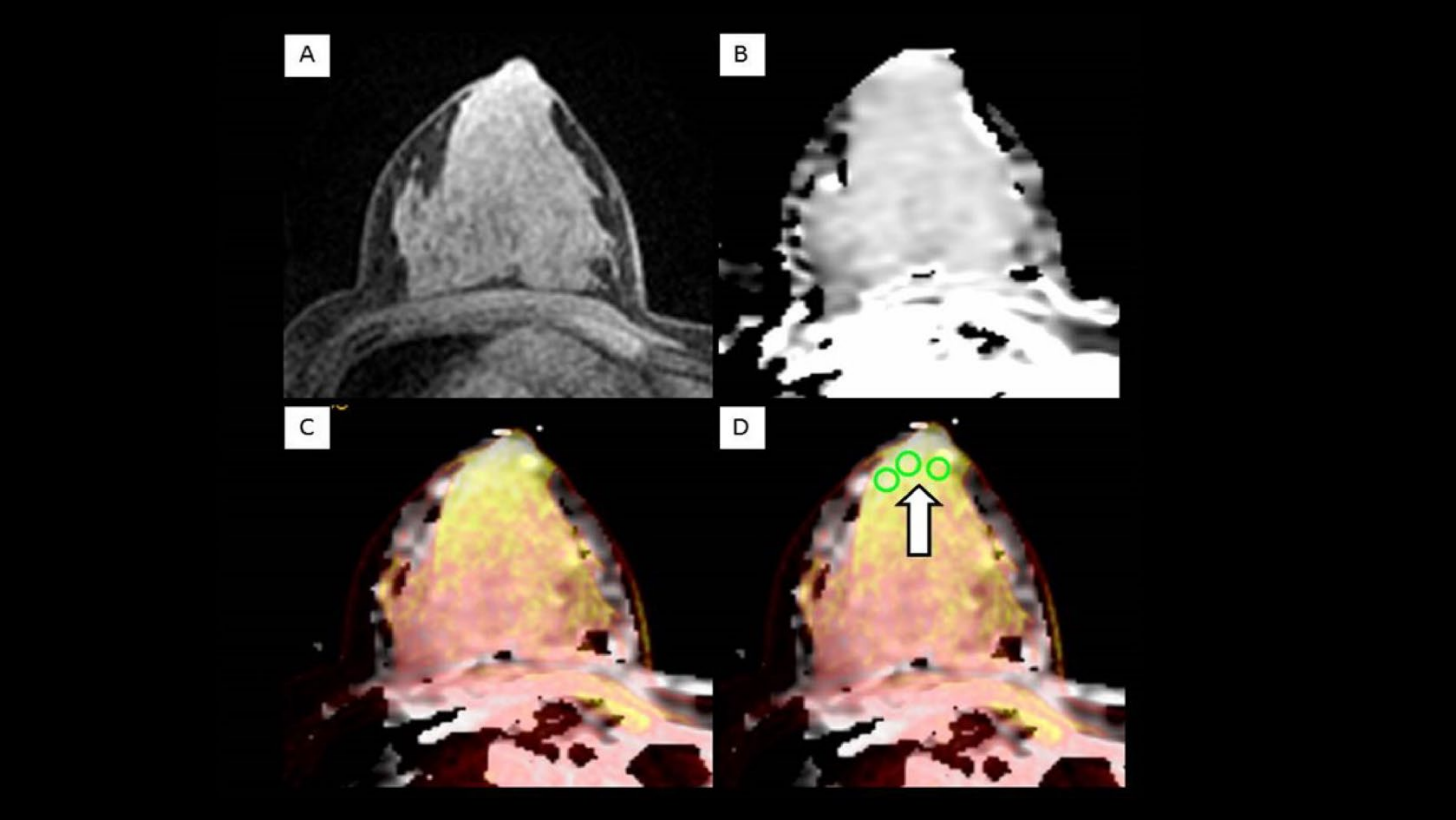
ROI placement for apparent diffusion coefficient (ADC) measurement in normal fibroglandular tissue. **(A)** T1-weighted fat-suppressed image. **(B)** Corresponding ADC map of the normal breast. **(C)** Fusion of T1-weighted fat-suppressed image and ADC map. **(D)** Single ROI placed in retroareolar fibroglandular tissue, avoiding ducts and vessels. ADC = apparent diffusion coefficient.

Imaging was performed on a 3.0T GE Signa HDx system using an 8-channel dedicated bilateral breast coil. Patients were positioned prone to ensure consistent breast geometry. The protocol included axial T2-weighted FSE, axial fat-suppressed STIR, and a DWI sequence with eight b-values (0, 50, 100, 200, 300, 400, 500, and 1000 s/mm²). DCE-MRI (3D spoiled gradient-echo) was acquired pre- and post-gadolinium (gadopentetate dimeglumine, 0.2 mL/kg) solely to serve as an anatomical reference for region of interest (ROI) placement. ROI placement for ADC measurement is illustrated in Fig. 1, and ROI placement in normal fibroglandular tissue is shown in Fig. 2.

### Image analysis and parameter calculation

Tumour ROIs (60–80 mm²) were manually drawn on the solid, enhancing portion of the tumour, identified using the early-enhancement DCE phase and diffusion restriction on DWI. Great care was taken to avoid necrotic regions (high T2, no enhancement) and distortion-prone edges. For patients with multifocal disease, the largest dominant lesion was analyzed. Normal fibroglandular tissue (FGT) ROIs were placed in the retroareolar region of the contralateral breast. ROI placement was performed by a breast radiologist with over 10 years of experience and reviewed by a second radiologist, with discrepancies resolved by consensus.

**Fig. 3:**
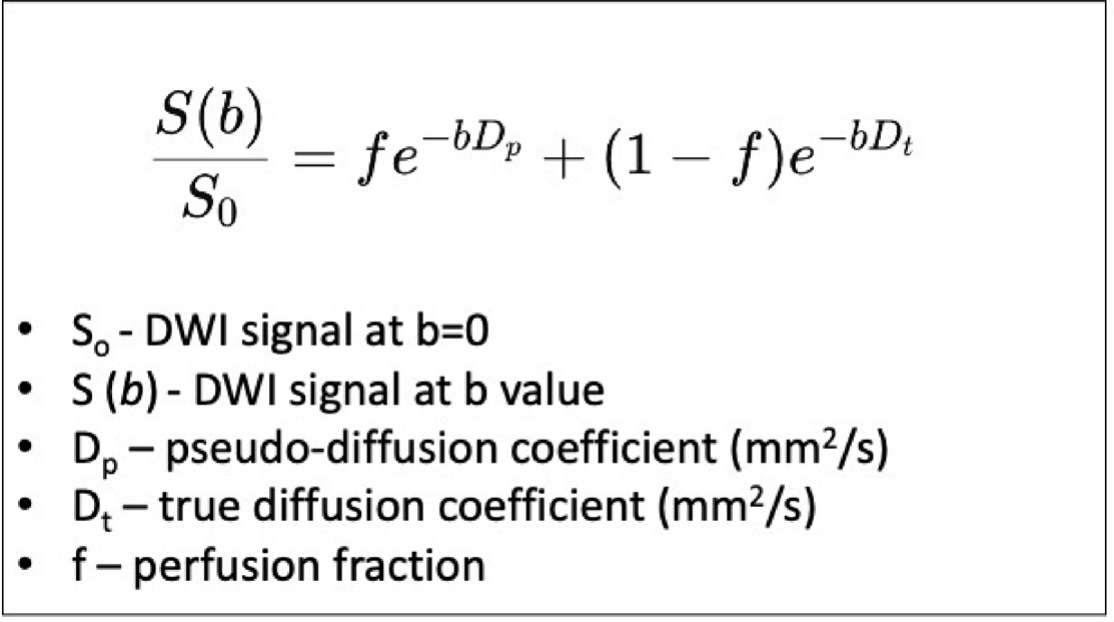
Bi-exponential intravoxel incoherent motion (IVIM) model used for parameter estimation. The model describes signal attenuation as a function of diffusion weighting (b-values), separating true molecular diffusion (Dt) from perfusion-related pseudo-diffusion (Dp) and perfusion fraction (f). S₀ represents the signal intensity at b = 0, and S(b) represents the signal at a given b-value.

**Fig. 4:**
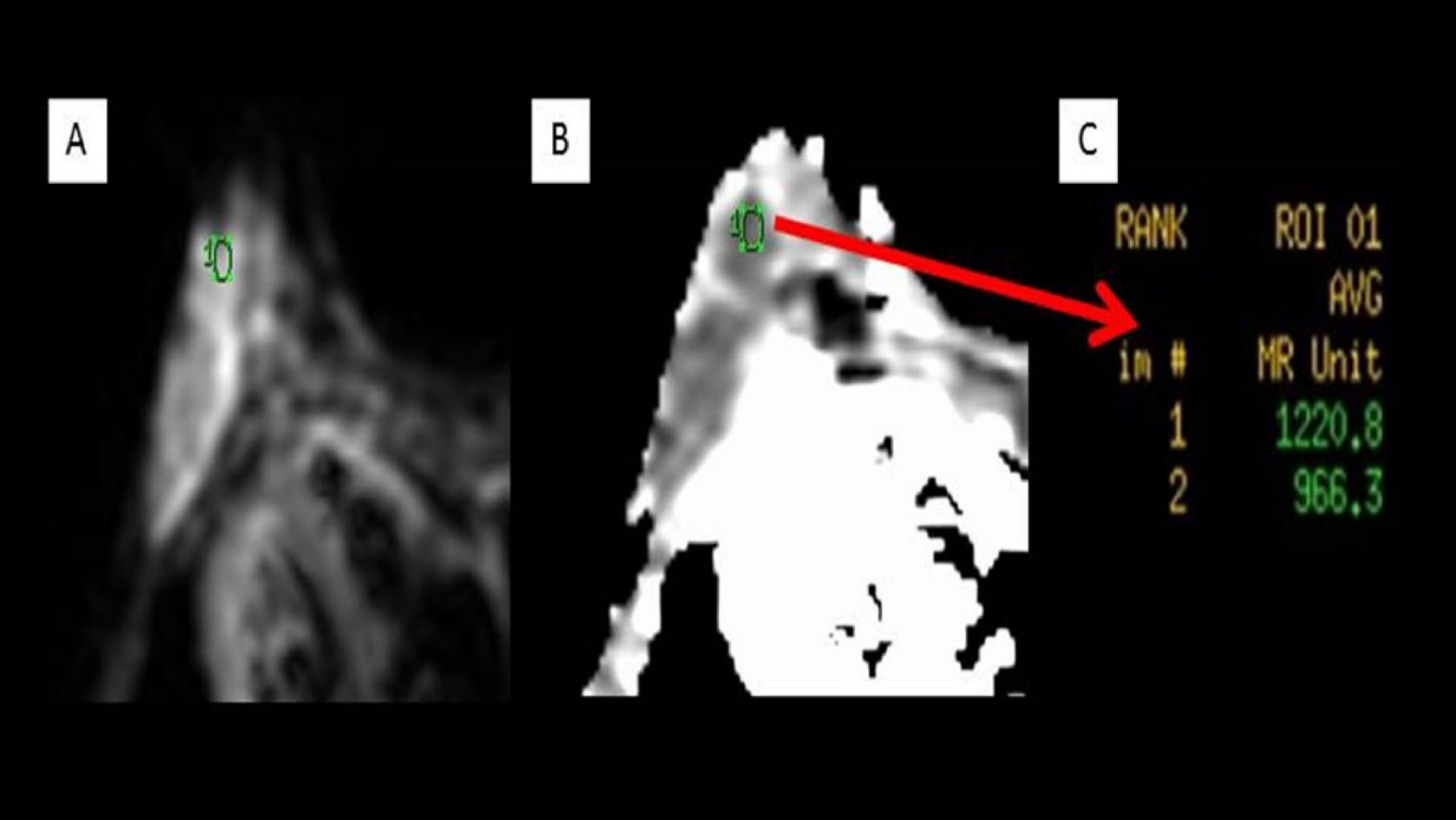
Post-processing workflow for IVIM parameter estimation. **(A)** Single ROI placement on diffusion-weighted imaging (DWI). **(B)** Corresponding ROI placement on the ADC map. **(C)** Signal intensities extracted across all b-values (50–1000 s/mm²) and fitted using a bi- exponential model to derive Dt, Dp, and f parameters. DWI = diffusion-weighted imaging; ADC = apparent diffusion coefficient.

ADC values were calculated using a mono-exponential model based on b = 0 and b = 1000 s/mm². IVIM parameters (Dt, Dp, and f) were estimated using a full bi-exponential model of the form S(b)/S₀ = f e^(− bDp) + (1 − f)e^(− bDt), where S₀ represents the signal intensity at b = 0 and S(b) represents the signal at each b-value. Parameter estimation was performed using a nonlinear least-squares fitting algorithm (Levenberg–Marquardt) applied across all eight b-values. Signal extraction was performed using OsiriX MD, and fitting was conducted using institutionally validated software. The bi-exponential IVIM model and post-processing workflow are illustrated in Figs. 3 and 4.

### Statistical analysis

Normality was assessed using the Shapiro–Wilk test. Longitudinal changes in tumour size (measured on T2-weighted images), ADC, and IVIM parameters were evaluated using paired t-tests for normally distributed data or Wilcoxon signed-rank tests for non-normal data. ROC curve analysis was performed to evaluate baseline diagnostic performance. A p-value ≤ 0.05 was considered statistically significant. Analysis was performed using SPSS version 23.0.

## Results

### Patient characteristics and tumour response

Fourteen women (mean age 43.9 years) with invasive ductal carcinoma (IDC) were enrolled. The cohort included eleven Grade III tumours, one Grade II, and two Grade I tumours. Five patients (35.7%) had triple-negative breast cancer (TNBC). All 14 patients completed MRI scans at baseline (T0) and after the first cycle (T1), while 4 patients completed the scan after the third cycle (T2). Attrition at T2 was due to treatment-related toxicity or patient withdrawal; analyses were performed using all available datasets at each timepoint. Following the initiation of chemotherapy, mean maximum tumour diameter decreased significantly from 5.25 cm at baseline (T0) to 3.78 cm after the first cycle (T1; *p* < 0.01) and 2.58 cm after the third cycle (T2; *p* < 0.01), representing a 28.0% and 50.9% size reduction, respectively (Table 1).

### Longitudinal diffusion and IVIM parameters

At baseline, malignant lesions demonstrated significantly lower diffusion values compared to normal fibroglandular tissue. Mean tumour ADC was 0.81 ± 0.11 × 10^− 3^ mm²/s compared to 1.90 ± 0.15 × 10^− 3^ mm²/s for normal tissue (*p* < 0.01). Similarly, true diffusion (*D*_*t*_) was significantly restricted in tumours (0.64 ± 0.09 × 10^− 3^ mm²/s) compared to normal tissue (1.51 ± 0.16 × 10^− 3^ mm²/s; *p* < 0.01).

Following chemotherapy, both diffusion parameters showed a progressive increase, mirroring the reduction in tumour size. Mean tumour ADC increased to 1.03 ± 0.14 × 10^− 3^ mm²/s at T1 (*p* < 0.01 vs. T0) and showed a further increase to 1.13 ± 0.20 × 10⁻³ mm²/s at T2. *D*_*t*_ followed a similar trajectory, increasing to 0.91 ± 0.12 × 10⁻³ mm²/s at T1 and remaining elevated at 0.98 ± 0.17 × 10⁻³ mm²/s at T2 (Table 1). Perfusion-related IVIM parameters (*D*_*p*_ and *f*) exhibited high variability and demonstrated no statistically significant differences between malignant and normal tissue or across treatment timepoints. Due to the limited number of patients completing the T2 examination (*n* = 4), statistical comparisons at T2 were considered exploratory and are presented descriptively.

**Table 1:** Longitudinal changes in tumour size and diffusion parameters during neoadjuvant chemotherapy.

Parameter	Baseline (T0) (*n* = 14)	Cycle 1 (T1) (*n* = 14)	Cycle 3 (T2) (*n* = 4)	*p*-value (T0 vs. T1)
**Tumour Size (cm)**	5.25	3.78	2.58	**< 0.01**
**ADC (x10** ^**− 3**^ **mm²/s)**	0.81 ± 0.11	1.03 ± 0.14	1.13 ± 0.20	**< 0.01**
***D***_***t***_ **(x10**^**− 3**^**mm²/s)**	0.64 ± 0.09	0.91 ± 0.12	0.98 ± 0.17	**< 0.01**
***D***_***p***_ **(x10**^**− 3**^**mm²/s)**	15.2 ± 6.1	16.0 ± 7.3	13.5 ± 5.8	0.68
***f*** **(%)**	7.8 ± 2.4	8.1 ± 2.7	7.5 ± 2.1	0.52

Note: Data presented as Mean ± Standard Deviation.

*D*_*t*_: True diffusion; *D*_*p*_: Pseudo-diffusion; *f*: Perfusion fraction.

### Subgroup and diagnostic analysis

In the subgroup of five TNBC patients, no distinct association was observed between molecular subtype and the magnitude of parameter changes, although the small sample size precludes definitive statistical comparison. ROC analysis indicated that baseline ADC achieved an area under the curve (AUC) of 1.00 (Cut-off: 1.52 × 10^− 3^ mm²/s) for differentiating IDC from normal tissue. However, this perfect separation is likely attributable to the clear contrast between high-grade tumours and normal background tissue in this small cohort and should be interpreted with caution.

## Discussion

This pilot study evaluated diffusion-weighted and intravoxel incoherent motion (IVIM) MRI parameters as potential non-contrast imaging biomarkers of neoadjuvant chemotherapy (NAC) response in women with locally advanced breast cancer (LABC). The principal findings were that both the apparent diffusion coefficient (ADC) and true diffusion (*D*_*t*_) increased following NAC, paralleling significant reductions in tumour size. These early microstructural changes reflect treatment-related decreases in cellular density and expansion of extracellular space and align with established diffusion imaging behaviour in responding breast tumours^21,22^. Although based on a small sample, the results support the feasibility of incorporating diffusion-derived indices into non-contrast MRI monitoring protocols for patients who cannot undergo repeated gadolinium-enhanced imaging.

Importantly, the role of diffusion and IVIM-derived parameters in this study is not to replace size-based assessment, but to provide complementary information regarding early microstructural changes that may precede or accompany tumour shrinkage. While tumour size reduction remains the clinical standard for response evaluation, it reflects macroscopic changes and may not capture early cellular response. Diffusion-based metrics such as ADC and Dt provide insight into tumour cellularity and extracellular space, which may change before measurable size reduction. In addition, these parameters may be particularly valuable in clinical scenarios where contrast-enhanced imaging is not feasible, thereby supporting the development of non-contrast MRI protocols for treatment monitoring.

At baseline, malignant lesions demonstrated significantly lower ADC and *D*_*t*_ values than contralateral fibroglandular tissue, consistent with previous reports showing restricted water diffusion in highly cellular breast cancers^23,24^. Following NAC, both parameters increased substantially by the first and third treatment cycles. These findings mirror results from larger trials—such as the ACRIN 6698 study—where increased ADC was strongly associated with treatment-related reductions in tumour cellularity^25^. Compared with ADC, *D*_*t*_ isolates pure molecular diffusion by separating perfusion contributions, potentially offering complementary information regarding tissue microstructure^13^. The observed parallel increases in ADC and *D*_*t*_ in our cohort strengthen confidence in their biological relevance^26^.

Perfusion-related IVIM metrics, including pseudo-diffusion coefficient (*D*_*p*_) and perfusion fraction (*f)*, did not show consistent differences between tumour and normal tissue nor clear longitudinal trends. This lack of reproducibility has been reported in several breast IVIM studies and may reflect the inherently low signal-to-noise ratio of perfusion-related components^27,28^. A recent study by Gong et al. emphasised that complex multi-b-value models require robust sample sizes and harmonised fitting to achieve stability^29^. Our findings suggest that while *D*_*p*_ and *f* are of investigational interest, diffusion-dominant metrics may presently be more reliable for treatment monitoring.

No significant associations were observed between triple-negative breast cancer (TNBC) status and parameter changes, likely due to the small subgroup size (*n* = 5). TNBC is known to exhibit heterogeneous treatment responses^30^, and recent meta-analyses suggest that molecular subtypes may display distinct diffusion signatures. Future studies with adequate power could explore whether high-grade or TNBC tumours demonstrate differential diffusion kinetics.

This study has several important limitations. The most significant is the attrition across MRI timepoints, with only four patients completing all three scans, which restricts the robustness of later-cycle analysis. Furthermore, tumour size changes alone may not fully capture early treatment effects, reinforcing the need for complementary imaging biomarkers. Additionally, ROI-based analysis captures only a portion of tumour heterogeneity compared to whole-tumour volumetric or histogram analysis. Despite these constraints, the study demonstrates the feasibility of obtaining diffusion parameters in a clinical LABC population and supports their potential value as non-contrast markers of early treatment response.

## Conclusion

This pilot study demonstrates that diffusion-weighted MRI and IVIM-derived parameters, particularly the apparent diffusion coefficient (ADC) and true diffusion (*D*_*t*_), exhibit measurable early changes during neoadjuvant chemotherapy in women with locally advanced breast cancer. These changes parallel tumour size reduction, reflecting treatment-related microstructural alterations and supporting the feasibility of diffusion-based metrics as non-contrast imaging biomarkers. These findings suggest that diffusion-derived parameters may provide complementary information to conventional size-based assessment, particularly in non-contrast imaging settings. Perfusion-related IVIM parameters (*D*_*p*_ and *f*) showed limited reliability under routine clinical acquisition conditions.

While these findings highlight potential clinical value in patients unable to undergo gadolinium-enhanced MRI, the pilot nature of the study and limited longitudinal datasets require cautious interpretation. Larger, standardised, pathology-correlated studies are needed to validate ADC- and *D*_*t*_-based monitoring strategies and to further define the role of IVIM MRI in neoadjuvant therapy assessment.

## Data Availability

The datasets generated and/or analysed during the current study are not publicly available due to institutional data protection policies but are available from the corresponding author upon reasonable request.
